# Effects of Green Manures (in the Form of Monoculture and Intercropping), Biofertilizer and Organic Manure on the Productivity and Phytochemical Properties of Peppermint (*Mentha piperita* L.)

**DOI:** 10.3390/plants11212941

**Published:** 2022-11-01

**Authors:** Abdollah Javanmard, Mostafa Amani Machiani, Mohammad Haghaninia, Luisa Pistelli, Basma Najar

**Affiliations:** 1 Department of Plant Production and Genetics, Faculty of Agriculture, University of Maragheh, Maragheh P.O. Box 55136-553, Iran; 2 Department of Pharmacy, University of Pisa, Via Bonanno Pisano, 12, 56126 Pisa, Italy; 3 Department of Agricultural, Food and Agro-Environmental Sciences, University of Pisa, Via del Borghetto, 80, 56124 Pisa, Italy

**Keywords:** arbuscular mycorrhizal fungi, medicinal and aromatic plants, menthol, secondary metabolites, sustainable agriculture

## Abstract

Nowadays, the application of sustainable and eco-friendly fertilizers plays an important role in improving the essential oil (EO) quantity and quality of medicinal and aromatic plants. Hence, the study aimed to investigate the effects of green manures, organic manures and biofertilizers on the nutrient content, dry matter yield, EO productivity and quality of peppermint. The treatments included green manures [barley monoculture (Bm), hairy vetch monoculture (HVm) and replacement intercropping of 75%HV + 25%B, 50%HV + 50%B and 25%HV + 75%B], arbuscular mycorrhizal fungi (AMF) and vermicompost (VC). A 50%HV + 50%B green manure appears to be the most effective treatment, since it provides the greatest amount of nutrients (N and K, 18.8 g kg^−1^, and 18.1 g kg^−1^, respectively), the tallest plants (61.5 cm), the most nodes per plant (17.5), the lateral branches (24.4), the highest leaf greenness index (45.5) and dry yield (266.7 g m^−2^) in peppermint. Moreover, this treatment evidenced the larger EO content (1.8%) and EO yield (4.84 g m^−2^). Regardless of the treatments, the chemical composition of peppermint EO was characterized by menthol (32.35–37.73%), menthone (16.96–20.64%) and 1,8-cineole (6.18–7.78%). The maximum content of menthol and 1,8-cineole was obtained by the application of 50%HV + 50%B. Additionally, the highest content of menthone was observed in AMF treatment. These results indicate that the application of 50%HV + 50%B green manures could be suggested as an environmentally friendly strategy for improving EO quantity and quality of peppermint.

## 1. Introduction

In recent years, more than 5000 plant species were investigated for chemical compositions with biological activities [1]. Among these, medicinal and aromatic plants are able to synthesize a diverse group of secondary metabolites with a broad range of pharmacological and therapeutic potentials and also are used in the cosmetic and food industries [2]. Peppermint (*Mentha piperita* L.) is a perennial medicinal and aromatic plant belonging to the Lamiaceae family, which is a natural hybrid of water mint (*Mentha aquatic* L.) and spearmint (*Mentha spicata* L.) [3]. By extracting more than 14,000 tons of EO from peppermint, this plant ranks as the second commercially most important EO in the world after citrus [4]. The EO of peppermint has biological activities such as antimicrobial, antibacterial, antifungal, antimutagenic and antidiabetic [5]. Additionally, the plants are used for treatment of coughs and colds, sinus infections, headaches, reducing pain, improving mental function and decreasing stress [2,6]. It has been reported that the terpenoids compounds such as menthol, menthone, 1,8-cineole, germacrene D and (*E*)-caryophyllene are the main EO constituents of peppermint [4,7].

In the modern agriculture systems, the increasing productivity of plants was achieved by excessive application of chemical inputs such as chemical fertilizers, pesticides and etc. [8]. The application of chemical inputs, in addition to hazardous effects on the environmental resources and human health, has negative impacts on the EO quality and bioactive compounds of medicinal and aromatic plants [1]. Therefore, the replacement of synthetic inputs with eco-friendly inputs such as green manures, organic manures and biofertilizers in the cultivation of medicinal and aromatic plants seems necessary.

In recent years, the use of green manure has received attention due to its environmentally friendly properties and its positive effects in improving soil health and the quality and quantity of plants [9]. Green manure is one of the oldest methods of agricultural management which involves the use of nutrient-rich plants, mainly including grasses or legumes [10]. The usage of green manures in sustainable agriculture systems has varied benefits, including decreasing runoff and soil erosion, increasing the content of macro and micro-nutrients and soil organic matter, decreasing the soil bulk density and also enhancing soil microbial and biological activity [11]. The use of leguminous plants as green manure is common in sustainable agricultural systems due to the biological nitrogen fixation and increasing the N availability in soils [10,11]. It is worth noting the increasing plant diversity as green manures enhance the efficiency of these crops in increasing nutrient accessibility, organic matter and soil health. One of the strategies to increasing the diversity of plants in green manure cultivation is intercropping, in which two or more species are grown contemporaneously in the same area during a growing season [12]. It has been reported that the intercropping of legumes with cereal as green manures improves soil fertility and improves the nutrients’ availability, which leads to increased plant productivity [13].

In addition to using green manures in sustainable agricultural systems, the application of organic manures (such as vermicompost) [14] and biofertilizers (such as arbuscular mycorrhizal fungi) can positively affect the plant’s productivity and its quality [15]. Arbuscular mycorrhizal fungi (AMF) can establish symbiotic association with more than 80% of the plant’s roots and affect the plant’s performance through improving nutrient accessibility, especially P and other micro-nutrients such as Zn and Fe, increasing water uptake and promoting the photosynthesis rate and also enhancing plant tolerance in the face of stressful conditions [16,17]. It has been reported that the inoculation of different AMF species improves the EO quality and quantity of medicinal and aromatic plants [18]. Rezaei-Chiyaneh et al. [16] reported that the inoculation of two AMF species (*Funneliformis mosseae* + *Rhizophagus irregularis*) improved the EO quantity and quality of black cumin (*Nigella sativa* L.) through increasing the main EO constituents including geranyl acetate, thymol, *p*-cymene, borneol and *trans*-caryophyllene.

Vermicompost (VC) is a nutritive organic fertilizer rich in NKP and other micro-nutrients that affected plant’s performance through the gradual releasing of nutrients in the plant growth period [19]. Due to VC’s ability to increase nutrient availability, increasing soil biological activity and soil health, the mentioned organic manure was used for increasing plant’s productivity, especially in low-input conditions [14]. It has been reported that VC acts as an organic soil conditioner that improves the quantity and quality characteristics of medicinal and aromatic plants [20]. Amooaghaie and Golmohammadi [21] noted that the application of VC increased chlorophyll content, photosynthesis rate and the EO content of thyme (*Thymus vulgaris* L.) seedlings.

Considering the negative effects of chemical fertilizers on the bioactive compounds of medicinal and aromatic plants, improving the quantity and quality characteristics of these plants, especially in low-input conditions, has become a major challenge in the agricultural sector. Literature is scant on the potential of green manures (in monoculture and intercropping conditions), AMF and VC applications for improving peppermint EO quantity and quality. Therefore, the study aimed to investigate the effectiveness of the mentioned fertilizers on the nutrient content, morphological and phytochemical characteristics of peppermint.

## 2. Results

### 2.1. Green Manures Productivity

**Figure 1:**
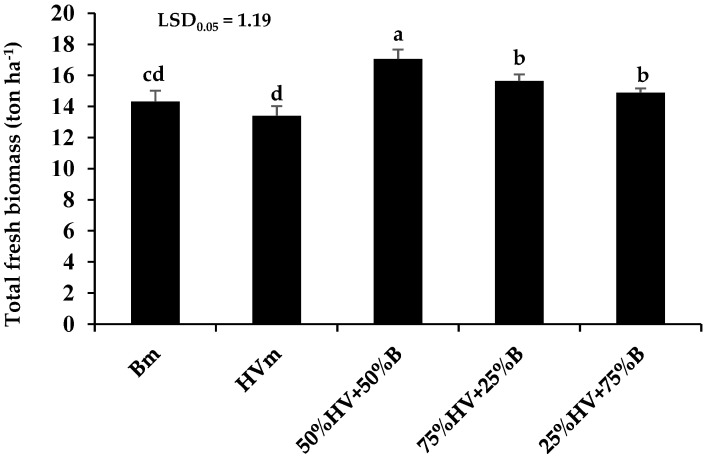
The total fresh biomass of green manures that returned into soil at flowering stage (average of two years). Bm (Barley monoculture); HVm (hairy vetch monoculture); 50%HV + 50%B, 75%HV + 25%B and 25%HV + 75%B (different intercrop ratios of hairy vetch/barley). Same letters above the bars do not differ significantly (*p* < 0.05) according to LSD test.

Among different green manures, the highest total fresh biomass yield (17.1 ton ha^−1^) was obtained in the 50%HV + 50%B intercropping. Moreover, the lowest total fresh biomass belonged to hairy vetch (HVm) and barley monoculture (Bm). The total fresh biomass in intercrops of 25%HV + 75%B, 50%HV + 50%B and 75%HV + 25%B was enhanced by 3.9, 19.1 and 9.3% in comparison with Bm and was enhanced by 11, 27.2 and 16.8% in comparison with HVm, respectively (Figure 1).

### 2.2. Peppermint

#### 2.2.1. Nutrient

**Figure 2:**
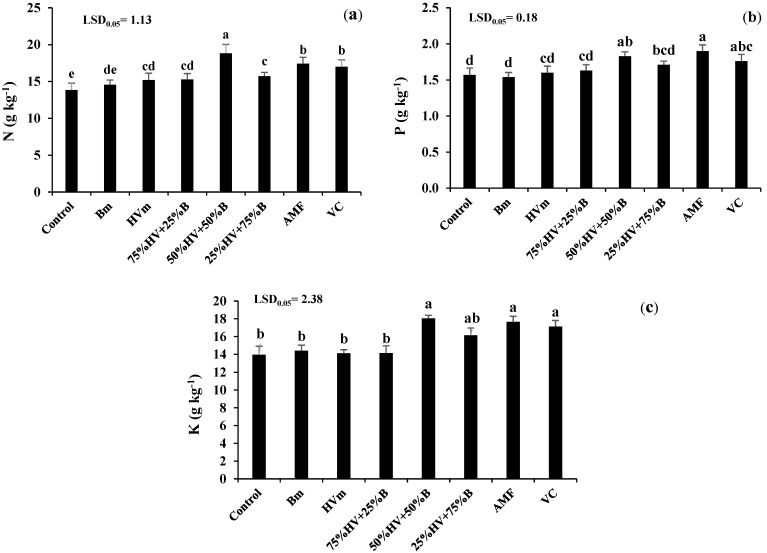
The content of N (**a**), P (**b**) and K (**c**) of peppermint in different fertilizer sources. Bm (Barley monoculture); HVm (hairy vetch monoculture); 50%HV + 50%B, 75%HV + 25%B and 25%HV + 75%B (different intercrop ratios of hairy vetch/barley); AMF (arbuscular mycorrhizal fungi); VC (vermicompost). Similar letters above the bars do not differ significantly (*p* < 0.05) according to LSD test.

The maximum content of N (18.8 g kg^−1^) and K (18.1 g kg^−1^) in peppermint was recorded in green manure intercropping at the ratio of 50%HV + 50%B, which was 36.1 and 29.3% higher than the control, respectively. Moreover, the highest content of P (1.9 g kg^−1^) belonged to the AMF application followed by 50%HV + 50%B (1.8 g kg^−1^). The P content of peppermint increased by 1.9, 3.8, 16.6, 8.9, 21 and 12.1% by the application of HVm, 75%HV + 25%B, 50%HV + 50%B, 25%HV + 75%B, AMF and VC, respectively (Figure 2a–c).

#### 2.2.2. Agronomic Traits

Among different fertilizer sources, the highest plant height (61.5 cm), number of nodes per plant (17.5) and number of lateral branches per plant (24.4) were achieved by the green manure intercrop at the ratio of 50%HV + 50%B which was 36.2, 78.2 and 43.4% higher than the control, respectively. Moreover, the highest number of leaves per plant (737) was measured in the AMF application, which was not significantly different with 50%HV + 50%B. It is worth noting that the lowest value of the above-mentioned traits was achieved in the control (non-application of fertilizer) (Table 1).

#### 2.2.3. Leaf Greenness

The maximum leaf greenness (45.5) was obtained in 50%HV + 50%B. The leaf greenness of peppermint was enhanced by 2.9, 3.8, 23, 30.3, 15.8, 27 and 21.3% by the application of Bm, HVm, 75%HV + 25%B, 50%HV + 50%B, 25%HV + 75%B, AMF and VC, respectively (Table 1).

#### 2.2.4. Dry Yield

Application of 50%HV + 50%B green manures was produced the highest dry yield of peppermint (266.7 g m^−2^), while the lowest dry yield (194.4 g m^−2^) was obtained in the control conditions. The application of Bm, HVm, 75%HV + 25%B, 50%HV + 50%B, 25%HV + 75%B green manures, AMF and VC enhanced the dry matter yield of peppermint by 4.6, 12, 23.9, 37.2, 28.3, 33.5 and 29.4%, when compared with non-fertilization, respectively (Table 1).

#### 2.2.5. Essential Oil Content

**Figure 3:**
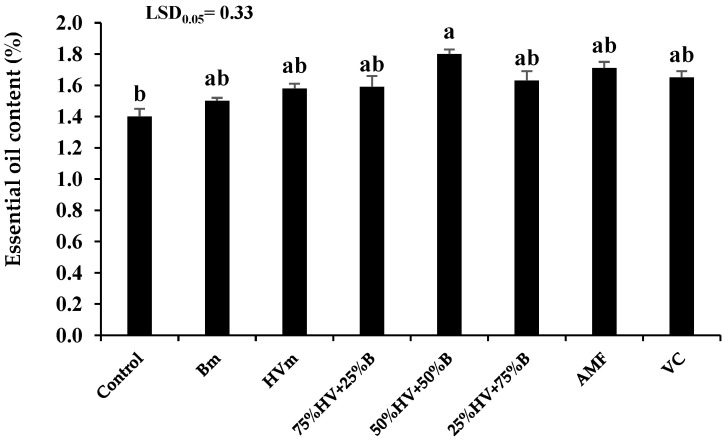
The essential oil content of peppermint in different fertilizer sources. Bm (Barley monoculture); HVm (hairy vetch monoculture); 50%HV + 50%B, 75%HV + 25%B and 25%HV + 75%B (different intercrop ratios of hairy vetch/barley); AMF (arbuscular mycorrhizal fungi); VC (vermicompost). Same letters above the bars do not differ significantly (*p* < 0.05) according to LSD test.

The maximum EO content of peppermint (1.8%) was achieved by the application of 50%HV + 50%B green manure. However, the lowest EO content of peppermint (1.4%) was observed in the non-application of fertilizer. The application of Bm, HVm, 75%HV + 25%B, 50%HV + 50%B, 25%HV + 75%B green manures, AMF and VC enhanced the EO content of peppermint by 7.1, 12.9, 13.6, 28.6, 16.4, 22.1 and 17.9%, when compared with non-fertilization (control), respectively (Figure 3).

#### 2.2.6. Essential Oil Yield

**Figure 4:**
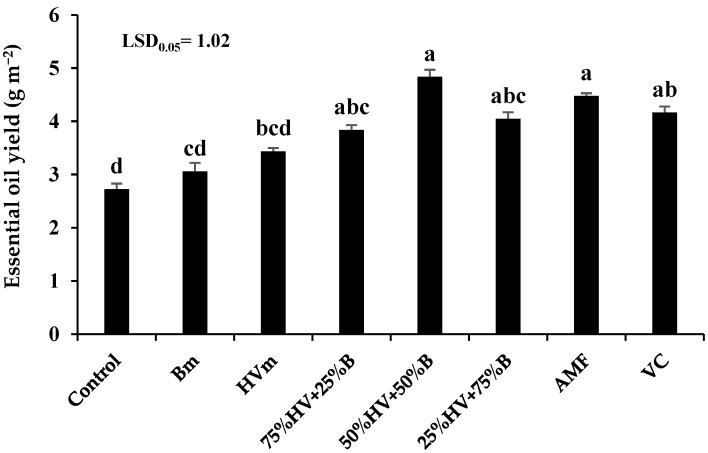
The essential oil yield of peppermint in different fertilizer sources. Bm (Barley monoculture); HVm (hairy vetch monoculture); 50%HV + 50%B, 75%HV + 25%B and 25%HV + 75%B (different intercrop ratios of hairy vetch/barley); AMF (arbuscular mycorrhizal fungi); VC (vermicompost). Same letters above the bars do not differ significantly (*p* < 0.05) according to LSD test.

Similar to the EO content, application of 50%HV + 50%B green manure produced the highest EO yield of peppermint (4.84 g m^−2^). In contrast, the lowest EO yield (2.73 g m^−2^) was achieved in the control (non-fertilization). The application of Bm, HVm, 75%HV + 25%B, 50%HV + 50%B, 25%HV + 75%B green manures, AMF and VC enhanced the EO yield of peppermint by 12.1, 26, 40.7, 77.3, 48.3, 64.1 and 52.7%, when compared with non-fertilization, respectively (Figure 4).

#### 2.2.7. Essential Oil Compositions

Based on the GC–MS and GC–FID analysis, 29 constituents were identified in peppermint EO, with the major constituents being menthol (32.35–37.73%), menthone (16.96–20.64%), 1,8-cineole (6.18–7.78%) and germacrene D (3.06–3.36%), respectively. The maximum content of menthol and 1,8-cineole was obtained by the application of 50%HV + 50%B green manures, which was 16.6 and 25.9% higher than non-fertilization (control). Additionally, the highest content of menthone and germacrene D was achieved in the AMF inoculation. It is worth noting that the lowest content of the four mentioned constituents was obtained in the control (non-fertilization) (Table 2).

## 3. Discussion

The obtained results demonstrated that the total fresh biomass yield among different cropping patterns of green manures was achieved in the intercrop of 50%HV + 50%B. It seems that increasing nutrient accessibility by nitrogen fixation of legume species (hairy vetch) and improving the environmental use efficiency in the intercropping patterns such as light, nutrient, water and etc. affect positively the total fresh biomass yield in comparison with plants monoculture [22,23]. Similarly, Miyazawa et al. [24] noted that the dual and triple intercropping of green manures improved nutrient uptake and enhanced the productivity in comparison with plant monoculture.

Our results showed that the N and K content of peppermint increased sharply in the intercrop of 50%HV + 50%B green manures. The increasing nutrient content of peppermint by the application of intercrop green manures could be explained by higher nutrient availability through improving the environmental use efficiency as a result of different root distribution of intercropping components and enhancement of nutrient uptakes at different depths of the soil [25]. Moreover, in the HV/B intercrop, the N availability of B was enhanced due to fixing a considerable quantity of N by the legume species (HV) [26]. Additionally, in intercropping patterns, the N and P content of the legume species at the early stage of plant growth were higher than the companion plants. After that, the nutrient content of the companion plant (B) improved by enhancing the N fixation rate, the root exudation of the legume species and also decreasing the soil pH through production of H^+^ [12]. Moreover, the content of P in peppermint was enhanced by the AMF inoculation. The AMF enhanced the solubility of P from less bioavailable P-minerals through extensive underground extra-radical mycelia and also the production of inorganic acids (carbonic acid and sulfuric acid), organic acids (oxalic, citric and lactic) and phosphatase enzymes [27]. Similarly, Amani Machiani et al. [25] noted that the inoculation of AMF (*Funneliformis mosseae*) enhanced the P content of *Thymus vaulgaris* L. by 31%.

The highest SPAD index was obtained in the 50%HV + 50%B green manures. The content of chlorophyll production in plants depends on the presence of macro- and micro-nutrients such as N, Mg and Fe; therefore, increasing the chlorophyll and leaf greenness could be attributed to higher accessibility of nutrients after application of 50%HV + 50%B green manures [28]. Bilalis et al. [29] reported that the application of red clover (*Trifolium pretense* cv. Nemaro) and vetiver (*Vicia sativa* cv. Alexandros) green manures increased the tobacco (*Nicotiana tabaccum*) SPAD index by 133.6 and 91.1%, respectively.

The agronomic traits of peppermint, such as plant height, number of nods per plant, number of leaves per plant, number of lateral branches per plant as well as peppermint dry yield were increased sharply in 50%HV + 50%B green manures. It can be concluded that the higher nutrient availability in the soil by the application of 50%HV + 50%B green manures affect cell division and enlargement as well as the chlorophyll content, the photosynthesis rate which led to improving plant productivity [30,31].

The EO content and yield of peppermint was the highest by application of 50%HV + 50%B green manures. The EO productivity in the medicinal and aromatic plants depend on the development and division of the glandular trichomes, EO channels and secretory ducts. It seems that the application of 50%HV + 50%B green manures enhanced the nutrient availability through the gradual releasing of nutrients and also decreasing the soil pH, which led to increasing the solubility of nutrients in the root rhizosphere zone [32,33]. Therefore, the enhancement of the EO content by the application of 50%HV + 50%B green manures could be explained by the role of the mentioned fertilizer in improving nutrient accessibility and increasing the number of EO secreting glands in peppermint. Similarly, Bidgoli et al. [34] reported that the application of green manure increased the EO productivity of peppermint by 23%. The EO yield depends on the plant’s dry yield and EO productivity. Therefore, the increasing EO yield of peppermint plant after the application of 50%HV + 50%B green manures attributed to the positive effect of the mentioned fertilizers in increasing the peppermint dry yield and EO content. Similar to our results, Singh et al. [35] reported that the application of cowpea (*Vigna unguiculata* L. Walp.) green manure enhanced the EO yield of peppermint and Palmarosa (*Cymbopopogon martinii* (Roxb.) Wats. var motia Burk.) by 25.2 and 17.7%, respectively.

In this study, the main EO constituents of peppermint such as menthol and 1,8-cineole were enhanced after application of 50%HV + 50%B green manures. The EO constituents are synthesized in the methyleritrophosphate and mevalonic acid pathways [36]. In these pathways, the availability of nutrients such as N and P through the application of green manures can increase the intermediate compounds of EO biosynthesis such as NADPH, ATP and Acetyl-CoA [25]. Moreover, the availability of P enhanced the precursor compounds of terpenoids such as isopentenyl pyrophosphate and dimethylallyl pyrophosphate, which play an important role in EO composition biosynthesis [37]. Dos Santos Marques et al. [38] noted that the EO quality of *Lippia alba* (Mill) N.E. improved by the application of velvet-bean (*Mucuna aterrima* Holland) as green manure. The authors noted that the main EO constituents of *L. alba* including *β*-myrcene, limonene, and carvone increased 1.3%, 4.2%, and 6.6% with velvet-bean.

## 4. Materials and Methods

### 4.1. Site Description

A three-year experiment (2018, 2019 and 2020) was conducted at the research farm of the Faculty of Agriculture, University of Maragheh, East Azerbaijan Province, Iran, with an altitude of 1477 m above sea level, longitude 46 degrees and 16 min east, and latitude 37 degrees and 24 min north. Before sowing, some soil samples were randomly selected in a depth of 0–30 cm for determining soil physical and chemical properties, which are shown in Table 3. Moreover, the meteorological data of the experimental area are reported in Table 4.

### 4.2. Experimental Design and Treatments

The treatments included non-application of fertilizer (control), green manures [barley monoculture (Bm, with density of 350 seeds per m^2^), hairy vetch monoculture (HVm; with density of 250 seeds per m^2^), and replacement intercropping of 75%HV + 25%B, 50%HV + 50%B and 25%HV + 75%B], arbuscular mycorrhizal fungi (AMF) and vermicompost (VC). In the replacement intercropping patterns, the required number of plants seeds (for example, 175 seeds per m^2^ of barley and 125 seeds per m^2^ of hairy vetch in 50%HV + 50%B) based on the density of the plant monoculture were mixed with others and sown. The experimental area was divided in two sections. In first section, the B and HV were sown as green manures in different cropping patterns (monoculture and intercropping) and turned into the soil at the flowering stage in the first year (2018) and the peppermint seedlings were sown in the second year (2019). The steps were repeated for a second time in 2019 and 2020 in the second section. In the AMF treatments, the *Rhizophagus intraradices* fungus (1000 spores/10 g soil) was isolated from the rhizosphere soil of the continuous cropping peppermint. For mass production of *R. intraradices*, spores were propagated in sterilized soil, river sand, and vermiculite cultured with alfalfa as a host plant for about 5 months in a greenhouse. AMF consists of *R. intraradices* colonized roots, spores, hyphae, and substrate [39]. To evaluate the number of spores, five samples (10 g) were randomly selected, and the number of spores were measured by Klironomos et al. [40] methods. In the VC treatments, 1.5 t ha^−1^ of manure was applied to the soil before planting. The VC used in this experiment was purchased from sayna-kesht company, Tehran, Iran, which was obtained using animal manure, beet leaves and other organic materials and a species of earthworm called *Eisenia foetida*. The chemical properties of VC are shown in Table 5. In order to determine the efficiency of experimental treatments, no additional fertilizers were used in this experiment. The barley and hairy vetch seeds were sown simultaneously on 20 March of 2018 and 2019, and peppermint rhizomes were planted in a depth of 3–5 cm on 10 April of 2019 and 2020. The number of rows, length of rows and distance between rows for the peppermint plants was set to be 5, 4 m and 45 cm, respectively. After planting, seedlings were watered using a drip irrigation system and the weeds were removed by hand as required.

### 4.3. Measurements

#### 4.3.1. The Fresh Biomass Yield of Green Manures

The aboveground biomass on B and HV was manually harvested at the flowering stage. After harvesting, the fresh biomass yield of each plot was recorded and reported as ton ha^−1^. Then, the harvested fresh biomass of the two plants was chopped (size 5–10 cm) and mechanically mixed into the surface soil (0–15 cm depth) [13].

#### 4.3.2. Peppermint

##### Dry Matter Yield

In both years, peppermint was harvested at 50% of the flowering stage on 28 July 2019 and 3 August 2020, respectively. Before harvesting, some growth characteristics of peppermint including plant height, number of leaves, number of nodes, and number of lateral branches were randomly measured in 10 samples from each treatment. To measure the dry matter yield of peppermint, 1 m^2^ of each plot was harvested randomly after removing the marginal effects.

##### Leaf Greenness Index (SPAD)

The leaf greenness index of peppermint was recorded from five leaves (middle part of the leaf blade) of each plot using SPAD 502, Minolta Ltd., Osaka, Japan [4].

##### Essential Oil Extraction and Analysis

The peppermint EO was extracted by the water distillation method using a Clevenger. For this purpose, 40 g of the aerial parts of peppermint were poured into the Clevenger and were added with 300 mL of distilled water and the extraction was performed at water boiling temperature for 3 h. After EO extraction, the required amount of sodium sulfate was added in samples and kept in a refrigerator (4 °C) in darkness for chemical analysis. The EO content and EO yield were calculated based on the following equations [6]:

Essential oil (%) = [EO extracted by hydro distillation/40 gr dried aerial parts] ×100

EO yield (g m^−2^) = EO content (%) × dry yield of aerial part (g m^−2^)

Moreover, the EO constituents were analyzed using GC–MS (GC–MS; 5977A, Agilent; Stevens Creek Blvd. Santa Clara, CA, USA) and GC–FID (Agilent 7990B; Stevens Creek Blvd. Santa Clara, CA, USA), following the previously method of Amani Machiani et al. [7].

##### Nutrient Concentration

The content of N, K and P in peppermint leaves was calculated based on the Kjeldahl method, flame photometry [41], and yellow method (using a spectrophotometer at 470 nm) [42], respectively.

### 4.4. Statistical Analysis

The normality and homoscedasticity of data regarding the morphological and physiological traits were verified using the Kolmogorov–Smirnov and Levene test, respectively. Data analysis was performed with SAS (version 9.3) software. Moreover, the significant differences among means were compared with the LSD test at *p* < 0.05.

## 5. Conclusions

The results of the study demonstrated that the intercropping of green manures (in the ratio of 50%HV + 50%B) improved nutrient uptake, morphological characteristics and dry matter yield of peppermint. Moreover, the EO content of peppermint and its quality improved by increasing the main EO constituents such as menthol and 1,8-cineole. Overall, it can be concluded that the intercropping of green manures could be suggested to peppermint growers as an alternative eco-friendly strategy of using chemical fertilizers for improving the EO quantity and quality of this plant.

## Figures and Tables

**Table 1:** The agronomic characteristics, leaf greenness, dry yield, essential oil content and essential oil yield of peppermint under different fertilizer sources (Average of two years).

Treatments	Plant Height (cm)	Number of Nodes per Plant	Number of Leaves per Plant	Number of Lateral Branches per Plant	Leaf Greenness (SPAD Index)	Dry Yield (g m^−2^)
**Control**	45.1 e	9.8 d	478.7 d	17.1 e	34.9 c	194.4 d
**Bm**	47.3 e	9.8 d	511.3 d	18.1 de	35.9 c	203.3 cd
**HVm**	50.4 d	11.7 c	545 d	19.8 cde	36.3 bc	217.7 c
**75%HV + 25%B**	50.6 d	12.2 c	625.8 c	21.6 abc	43 a	240.8 b
**50%HV + 50%B**	61.5 a	17.5 a	706.3 ab	24.4 a	45.5 a	266.7 a
**25%HV + 75%B**	54.1 c	12.1 c	685.8 abc	22.6 abc	40.5 abc	249.3 ab
**AMF**	58.6 b	16.9 a	737 a	23.3 ab	44.4 a	259.5 ab
**VC**	55.5 c	15 b	658.8 bc	20.4 bcd	42.4 ab	251.7 ab
**LSD**	2.51	1.86	69.58	3.15	6.31	19.84

Bm (Barley monoculture); HVm (hairy vetch monoculture); 50%HV + 50%B, 75%HV + 25%B and 25%HV + 75%B (different intercrop ratios of hairy vetch/barley); AMF (arbuscular mycorrhizal fungi); VC (vermicompost). Similar letters above the bars do not differ significantly (*p* < 0.05) according to LSD test.

**Table 2:** The essential oil constituents of peppermint under different fertilizer sources (average of two years).

							Treatments				
No.	Components	RI	RI lit	C	Bm	HVm	25%HV + 75%B	50%HV + 50%B	75%HV + 25%B	AMF	VC
1	*α*-Pinene	931	932	0.3	0.36	0.32	0.54	0.30	0.51	0.47	0.36
2	Sabinene	970	969	0.58	0.31	0.49	0.43	0.18	0.36	0.44	0.39
3	*β*-Pinene	975	974	0.79	0.55	0.8	0.71	0.79	0.72	0.84	0.92
4	Myrcene	988	988	0.46	0.39	0.47	0.32	0.46	0.41	0.40	0.72
5	3-octanol	1000	998	0.2	0.18	0.14	0.44	0.11	0.56	0.16	0.96
6	*α*-Terpinene	1017	1014	0.51	0.68	0.78	0.54	0.11	0.57	0.62	0.14
7	Limonene	1026	1024	2.91	0.84	1.16	2.92	1.91	1.31	1.82	2.52
8	1,8-cineole	1029	1026	6.18	6.24	6.69	6.31	7.78	6.47	6.91	6.33
9	*γ*-Terpinene	1058	1054	0.58	0.08	0.19	0.78	0.58	0.52	0.22	0.21
10	*cis*-Sabinene hydrate	1066	1065	1.37	0.93	1.00	1.21	1.09	0.96	1.23	1.2
11	Linalool	1103	1095	0.74	0.42	0.43	0.30	0.44	0.43	0.42	0.43
12	Menthone	1152	1148	16.96	18.61	19.53	17.32	19.26	17.42	20.64	19.05
13	Menthofuran	1161	1159	3.14	3.82	2.28	3.10	1.14	2.97	2.29	1.83
14	δ-Terpineol	1162	1162	3.49	3.46	3.65	3.76	3.94	3.13	3.79	3.64
15	*neo*-Menthol	1163	1161	3.18	3.25	3.71	3.39	3.81	3.88	3.52	3.22
16	Menthol	1175	1167	32.35	34.91	35.77	35.11	37.73	36.91	36.11	35.63
17	Terpinen-4-ol	1177	1177	0.06	0.26	0.14	0.70	0.22	0.88	0.19	0.13
18	*neo*-*iso*-Menthol	1184	1184	3.41	3.68	4.00	3.21	3.81	3.77	3.83	3.59
19	Pulegone	1236	1233	3.05	2.35	2.13	2.18	2.05	2.01	2.09	2.11
20	Piperitone	1252	1252	2.54	1.96	2.2	2.51	1.54	2.46	1.36	1.49
21	*neo*-Menthyl acetate	1273	1271	0.88	0.76	0.6	0.95	0.47	0.57	0.45	0.57
22	*p*-Menth-1-en-9-ol	1294	1294	2.36	1.97	1.41	1.42	1.16	1.89	2.17	1.65
23	*iso*-Menthyl acetate	1307	1304	0.47	0.37	0.3	0.15	0.24	0.23	0.19	0.3
24	*β*-Bourbonene	1382	1387	0.87	0.43	0.22	0.31	0.17	0.41	0.1	0.62
25	(*E*)-Caryophyllene	1416	1417	2.29	2.59	2.11	1.96	2.19	2.35	2.66	2.29
26	(*E*)-*β*-Farnesene	1457	1454	0.68	0.13	0.23	0.34	0.45	0.50	0.93	0.49
27	Germacrene D	1479	1484	3.06	3.44	3.13	3.28	3.24	3.36	3.35	3.16
28	Elixene	1494	1492	1.44	1.31	1.05	0.88	0.9	1.08	0.41	1.12
29	Viridiflorol	1589	1592	0.77	0.25	0.63	0.15	0.86	0.7	0.18	0.33
	Total identified (%)			95.62	94.53	95.56	95.22	96.93	97.34	97.79	95.4

Bm (Barley monoculture); HVm (hairy vetch monoculture); 50%HV + 50%B, 75%HV + 25%B and 25%HV + 75%B (different intercrop ratios of hairy vetch/barley); AMF (arbuscular mycorrhizal fungi); VC (vermicompost). RI, linear retention indices on DB-5 MS column, experimentally determined using homologue series of n-alkanes. RI lit, Relative retention indices taken from Adams.

**Table 3:** Soil analysis results before beginning the experiment (depth 0–30 cm).

Texture	N (%)	P (mg kg^−1^)	K (mg kg^−1^)	pH	EC (dS m^−1^)	Organic Matter (%)
(Sandy clay loam)	0.085	9.32	510	8.2	0.255	0.78

**Table 4:** Monthly average temperature and total monthly precipitation growing seasons.

Year	April	May	June	July	August	September	October
Monthly average temperature (°C)							
2018	12.6	16.6	24.1	30.2	27.7	23.6	15.9
2019	10.4	18.5	25.7	27.6	27.8	22.1	16.7
2020	11.8	19.1	24.2	28	25.1	23.8	16.1
Total monthly precipitation (mm)							
2018	44.9	54.5	1.7	0.1	0	0	14.5
2019	51.3	37.8	4.2	0	0	0	6.3
2020	63.3	12	2.6	0.1	1.2	0	0.4

**Table 5:** Chemical properties of the vermicompost fertilizer.

Total Nitrogen (%)	P (%)	K (%)	pH	EC (µmhos.cm^−1^)	Organic Carbon (%)	Cu (ppm)	Zn (ppm)	Ca (ppm)
1.6	0.47	0.27	7.84	1.64	7.13	3.84	66.6	8.75

## Data Availability

Not applicable.
